# Diabetic Complications and Factors Affecting Glycemic Control Among Patients With Type II Diabetes Mellitus Attending the Chronic Illness Clinics at Tabuk, Saudi Arabia

**DOI:** 10.7759/cureus.11683

**Published:** 2020-11-24

**Authors:** Shahad J Ghabban, Bashayr Althobaiti, Ibrahim M Farouk, Manea Al Hablany, Ahmad Ghabban, Rawabi Alghbban, Saleh Harbi, Asem E Albalawi

**Affiliations:** 1 Department of Family Medicine, King Salman Northwest Armed Forces Hospital, Tabuk, SAU; 2 College of Medicine, University of Tabuk, Tabuk, SAU

**Keywords:** diabetes mellitus, glycemic control, saudi arabia, complications, insulin

## Abstract

Background

Diabetes mellitus (DM) is one of the most common diseases worldwide and affects many patients all over the globe. Diabetic complications vary between microvascular and macrovascular, affecting a wide range of organs and systems in the body. Understanding, determining, and in return, preventing such factors is essential to alleviate the quality of life of diabetic patients. Therefore, we aim to determine the poor glycemic control and the factors associated with it in our diabetes population.

Methods

This is a retrospective study where data was reviewed for all patients with Type II DM (T2DM) who attended the chronic illness clinic at King Khaled Hospital in Tabuk. We included patients aged ≥ 18 years, of Saudi nationality, and residents of the Tabuk region. Any patient not satisfying all the inclusion criteria was excluded from the current study. Diagnosis with diabetes was made according to the American Diabetes Association guidelines, 2020. Patients’ demographic data, medical history, and social and lifestyle history were extracted from records. In addition, age, body mass index (BMI), drugs (insulin vs. oral hypoglycemic agents), duration of the disease, lipid profile, and other comorbidities were also extracted from the files. A p-value of <0.05 was selected as the statistically significant level in all tests.

Results

A total of 697 patients were included in the current study, with a mean age of 58.2±11.6 years. The mean glycosylated hemoglobin (HbA1c) of the study participants was 8.4±1.7%, and their fasting blood sugar (FBS) level was 9.9±3.9 mmol/l. With HbA1c cut-off at 7%, the overall prevalence of poor glycemic control was 81.5% (565/693). A higher prevalence of poor glycemic control was reported among study participants with higher DM duration (p=0.002). Diabetes complications were found in 208 (29.8%) of the study participants, where microvascular complications were present in 140 patients, and microvascular ones were found in 102. In the logistic regression model, older patients were less prone to poor glycemic control (OR=0.98; 95% CI=0.96-0.99; p=0.010). In addition, longer disease duration was a predictive factor of poor glycemic control (OR=1.05; 95% CI=1.02-1.08; p=0.003). Furthermore, the usage of combined insulin and tablet treatments were associated with a higher risk of poor glycemic control when compared to insulin only treatments (OR=4.65; 95% CI=1.55-13.94; p=0.006).

Conclusion

The results of this study indicate a high prevalence rate of poor glycemic control among Saudi patients, which is higher than previous reports have shown. More interest should be given to awareness programs with regard to promoting self-control protocols for the disease.

## Introduction

Diabetes mellitus (DM) is one of the most common diseases worldwide and affects many patients all over the globe. Additionally, the disease rates are rapidly growing, and it is now considered a world health emergency that puts a heavy burden on almost all countries [1]. An estimated 415 million adults have been diagnosed with diabetes, and this number is expected to rise to 642 million in the next two decades [2]. In 2013 alone, DM has killed 4.6 million patients worldwide [3]. The majority of DM patients are located in low and middle-income countries with prevalence rates of 77% and 88% for morbidity and mortality, respectively, in these countries [4]. A total of 3,852,000 cases of DM were recorded in Saudi Arabia in 2017, and therefore, it is now considered among the top ten countries with the highest numbers of DM cases [5]. The prevalence of DM in Saudi Arabia was estimated at 7% in 1989; however, the rate has hugely increased in the following decades, reaching 32% in 2009 [6]. Furthermore, DM has also been noticed to be rising among rural areas around the kingdom, with women twice as much as men [7]. Meanwhile, the government's financial burden is hefty, with an annual cost of diabetes has been estimated to be more than 0.87 billion dollars [8].

The etiology behind hazardous events of DM can be generally put down to poor glycemic control in these patients. Poor glycemic control will eventually lead to many micro and macro-vascular complications. Nevertheless, proper control of DM, which can be achieved by reducing the concentration of glycated hemoglobin (HbA1c) in the blood, can effectively reduce the development of these micro and macro-vascular complications [9]. Previous research has demonstrated that reducing HbA1c from 9.1 to 7.3% has effectively reduced the risk of developing a microvascular disorder by 41%, neuropathy by 60%, retinopathy by 63%, and nephropathy by 54%. It has also been estimated that any increase in the levels of HbA1c can eventually lead to an 18% and 30% increase in the rate of cardiovascular and microvascular events [10].

To achieve better glycemic control in DM patients, it is essential to determine the factors associated with glycemic control that will consequently lead to the prevention of complications and enhance the therapeutic approaches for DM. Many risk factors have been identified by previous studies worldwide [11, 12]. These factors will eventually lead to diminished pancreatic insulin secretion, insulin sensitivity, and action on the peripheral tissue [13]. The presence of comorbidities and chronic illnesses has been reported with poor glycemic control in addition to polypharmacy [14]. Despite the relatively large number of studies published on this subject, these factors are hugely variable among different countries and regions, which indicates the need for national investigations to identify the specific risk factors for their populations and achieve better prevention. Therefore, we aim to understand and determine the factors that affect glycemic control and explore various complications found along with it.

## Materials and methods

Study design

In this retrospective study data was reviewed for all patients with Type II DM (T2DM) who attended the chronic illness clinic at King Salman North West Armed Forces Hospital in Tabuk, Saudi Arabia. Diagnosis with diabetes was made according to the American Diabetes Association (ADA) guidelines, 2020 [15]. All patients satisfying the inclusion criteria were enrolled in the study. The inclusion criteria included an established diagnosis with T2DM, patients attending chronic illness clinics, aged ≥ 18 years, Saudi nationality, and residents of the Tabuk region. Any patient not satisfying all the inclusion criteria was excluded from the current study. 

Data collection

Clinical data of patients attending the chronic illness clinics at King Khaled Hospital in Tabuk between September 2019 and February 2020 were retrospectively reviewed. Patients’ demographic data, medical history, and social and lifestyle history were extracted from records. In addition, age, body mass index (BMI), drugs (insulin vs. oral hypoglycemic agents), duration of the disease, HbA1c, lipid profile, and data about other comorbidities were also extracted for all patients. Moreover, chronic complications that developed after the proper diagnosis of T2DM and that could be attributed to diabetes were considered in this study, including glomerular filtration rate (GFR), microalbuminuria, retinopathy, neuropathy, and atherosclerotic cardiovascular disease. The cut-off levels were used according to the ADA guidelines, 2020 [15].

Statistical analysis

Data was analyzed by using the Statistical Package for the Social Sciences (SPSS V.26, IBM Inc., Armonk, USA). Descriptive statistics (frequency, mean, and standard deviation) were calculated for all variables. Also, comparative analyses were carried out by chi-square test or unpaired t-test based on the type of variable. Furthermore, logistic regression was conducted with a calculating odds ratio (OR) to assess the association between glycemic control in T2DM and different possible risk factors. A p-value of <0.05 was selected as the statistically significant level in all the tests.

## Results

Sociodemographic characteristics

A total of 697 patients were included in the current study, with a mean age of 58.2±11.6 years. The age group of 45-64 years contained the most patients (58.5%). Most of the patients were males (63.7%), and the majority of them reported that they do not smoke tobacco (89.0%). The mean duration of DM disease was 10.8±7.3 years. Most patients (67.5%) were prescribed tablets only, 28.2% of patients were prescribed a combination of insulin and tablets, and insulin alone was given to only 4.3% of the patients. There was a statistically significant difference between treatment categories in terms of age groups (p=0.007) and the duration of the disease (p<0.001) as it was more likely for older age to have a higher duration of the disease (Table 1).

**Table 1:** Sociodemographic characteristics of diabetes mellitus patients (N=697) NA - not available; SD - standard deviation * Statistically significant at <0.05 level; ** Statistically significant at <0.001 level

Variables		Treatment categories								P-value
		Insulin only		Tablets only		Insulin and tablets		Total		
		n	%	n	%	n	%	N	%	
Age groups (years)	25–44	4	13.3	70	14.9	14	7.1	88	12.6	0.007*
	45–64	12	40.0	275	58.5	121	61.4	408	58.5	
	≥65	14	46.7	125	26.6	62	31.5	201	28.8	
Sex	Male	20	66.7	302	64.3	122	61.9	444	63.7	0.814
	Female	10	33.3	168	35.7	75	38.1	253	36.3	
Tobacco use	Yes	1	3.3	29	6.2	12	6.1	42	6.0	0.485
	No	29	96.7	420	89.4	171	86.8	620	89.0	
	NA	0	0.0	21	4.5	14	7.1	35	5.0	
Duration of disease (years); mean±SD		15.9	9.8	9.0	6.3	14.1	7.4	10.8	7.3	<0.001**

Prevalence of poor glycemic control

The data for HbA1c was only available for 693 of the included patients. The mean HbA1c of the study participants was 8.4±1.7%, and their fasting blood sugar (FBS) level was 9.9±3.9 mmol/l. With an HbA1c cut-off of 7%, the overall prevalence of poor glycemic control was 81.5% (565/693), whereas 18.5% of the patients showed good glycemic control (128/693).

Clinical and anthropometric measurements

A higher prevalence of poor glycemic control was reported among study participants with higher DM duration (p=0.002). In contrast, there was no statistically significant difference in poor glycemic control rates when it came to different BMI groups (p=0.504), systolic blood pressure (BP) (p=0.699), diastolic BP (p=0.400), low-density lipoproteins (LDL) levels in mg/dl (p=0.456), albumin-creatinine ratios (p=0.401), urine microalbumin (p=0.645), or estimated glomerular filtration rate (eGFR; p=0.208) (Table 2).

**Table 2:** Clinical and anthropometric measurements of diabetes mellitus patients (N=693) BMI - body mass index; BP - blood pressure; eGFR - estimated glomerular filtration rate; FBS - fasting blood sugar test; HbA1c - glycated hemoglobin; LDL - low-density lipoproteins; SD - standard deviation * Statistically significant at <0.05 level; ** Statistically significant at <0.001 level

Variables	Glycemic control						P-value
	Good n=128 (18.5%)		Poor n=565 (81.5%)		Total N=693 (100.0%)		
	Mean	SD	Mean	SD	Mean	SD	
Duration of disease (years)	9.0	6.8	11.2	7.3	10.8	7.3	0.002*
BMI	31.6	6.0	31.2	5.7	31.3	5.7	0.504
Systolic BP	134.4	17.8	133.7	15.7	133.8	16.1	0.699
Diastolic BP	69.0	10.1	69.8	9.1	69.6	9.3	0.400
FBS (mmol/l)	7.1	2.7	10.5	3.8	9.9	3.9	< 0.001**
HbA1c (%)	6.3	0.5	8.9	1.4	8.4	1.7	< 0.001**
LDL (mg/dl)	2.7	0.9	2.8	1.0	2.8	1.0	0.456
Albumin-creatinine ratio	189.3	637.7	98.4	314.4	112.0	379.9	0.401
Urine microalbumin	0.1	0.2	0.2	2.4	0.2	2.2	0.645
eGFR	90.6	25.5	95.3	39.3	94.4	37.1	0.208

Diabetes complications

**Figure 1:**
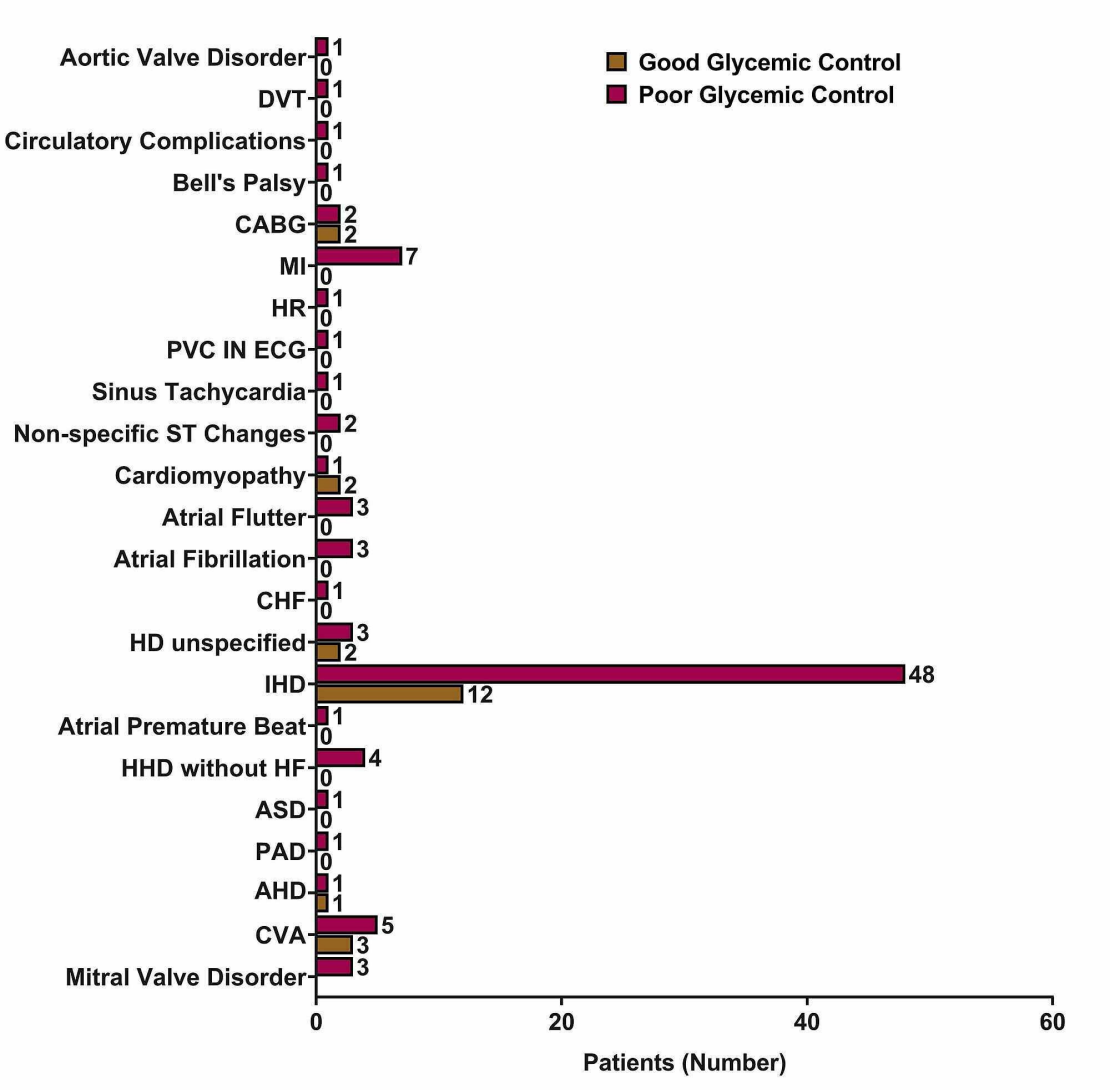
The distribution of macrovascular complications according to glycemic control AHD - atherosclerotic heart disease; ASD - atrial septal defect; CABG - coronary artery bypass grafting; CHF - congestive heart failure; CVA - cerebrovascular accident; DVT - deep venous thrombosis; ECG - electrocardiogram; HF - heart failure; HHD - hypertensive heart disease; IHD - ischemic heart disease; MI - myocardial infarction; PAD - peripheral arterial disease; PVC - premature ventricular complex

Diabetes complications were found in 208 (29.8%) of study participants, where microvascular complications were present in 140 patients, and macrovascular complications were found in 102 patients. Ischemic heart disease was the most common macrovascular complication (n=60), followed by cerebrovascular accidents (n=8), myocardial infarction (n=7), non-specified heart disease (n=5), and coronary artery bypass grafting. Figure 1 shows the distribution of macrovascular complications according to glycemic control.

**Figure 2:**
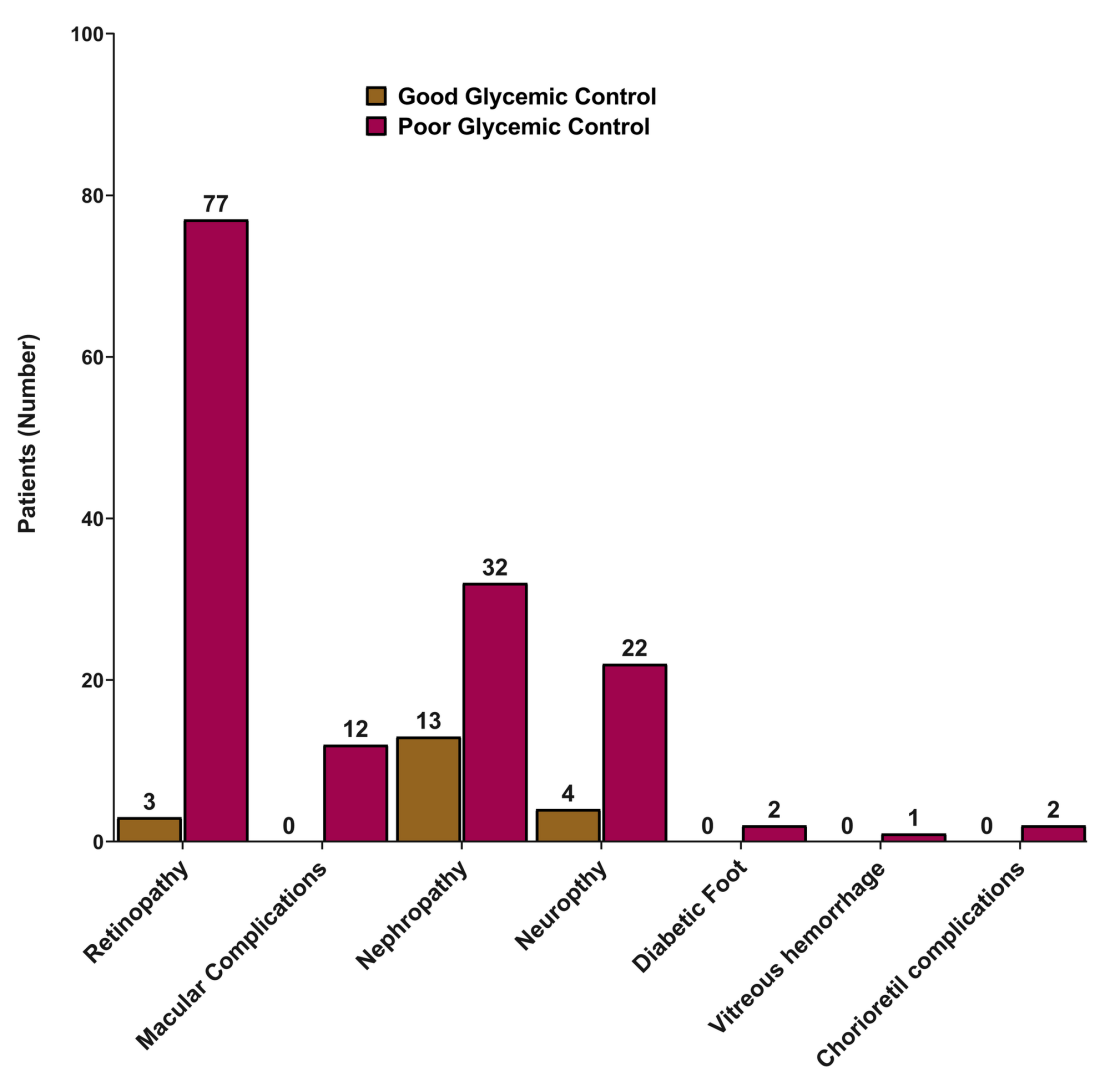
Distribution of microvascular complications according to glycemic control

In the same context, retinopathy was the common microvascular complication, which was found in 80 patients, followed by nephropathy (n=45), neuropathy (n=26), and macular complications (n=12). Figure 2 shows the distribution of microvascular complications according to glycemic control.

Factors associated with poor glycemic control

In the logistic regression model, older patients were less prone to poor glycemic control (OR=0.98; 95% CI=0.96-0.99; p=0.010). There was a significant increase in the risk of poor glycemic control in the 45-64 age group compared to the ≥65 age group (OR=1.55; 95% CI=1.02-2.37; p=0.041). Moreover, longer disease duration was a predictive factor of poor glycemic control (OR=1.05; 95% CI=1.02-1.08; p=0.003). Furthermore, the usage of combined insulin and tablet treatments was associated with a higher risk of poor glycemic control when compared to insulin only (OR=4.65; 95% CI= 1.55-13.94; p=0.006) (Table 3).

**Table 3:** Logistic regression analysis of factors associated with poor glycemic control among DM patients (N=693) BMI - body mass index; BP - blood pressure; LDL - low-density lipoproteins; SE - standard error; DM - diabetes mellitus * Statistically significant at <0.05 level

Predictor	Estimate	SE	P-value		95% confidence interval	
				Odds ratio	Lower	Upper
Age	-0.02	0.01	0.010*	0.98	0.96	0.99
Age groups (years)						
≥65	reference					
25–44	0.29	0.32	0.373	1.33	0.71	2.52
45–64	0.44	0.22	0.041*	1.55	1.02	2.37
Sex						
Male	reference					
Female	-0.15	0.2	0.459	0.86	0.58	1.28
Duration of disease (years)	0.05	0.02	0.003*	1.05	1.02	1.08
Tobacco use	0.12	0.43	0.784	1.12	0.49	2.6
BMI	-0.01	0.02	0.503	0.99	0.96	1.02
Systolic BP	0	0.01	0.676	1.00	0.99	1.01
Diastolic BP	0.01	0.01	0.400	1.01	0.99	1.03
LDL (mg/dl)	0.08	0.1	0.455	1.08	0.88	1.32
Treatment categories						
Insulin only	reference					
Tablets only	-0.23	0.47	0.620	0.79	0.32	1.99
Insulin and tablets	1.54	0.56	0.006*	4.65	1.55	13.94
Macrovascular complications	0.07	0.28	0.817	1.07	0.62	1.85
Microvascular complications	0.11	0.25	0.651	1.12	0.69	1.82

## Discussion

We conducted this study to identify the possible factors associated with poor glycemic control in diabetes patients. There was a significant increase in the risk of poor glycemic control in the 45-64 age group when compared to the ≥65 age group (OR=1.55; 95% CI=1.02-2.37; p=0.041). Moreover, longer disease duration was a predictive factor of poor glycemic control (OR=1.05; 95% CI=1.02-1.08; p=0.003). Meanwhile, microvascular complications were present in 140 patients, and macrovascular complications were found in 102 patients, with ischemic heart disease being the most common macrovascular complication (n=60), followed by cerebrovascular accidents (n=8), myocardial infarction (n=7), non-specified heart disease (n=5), and coronary artery bypass grafting. For this purpose, we have studied the association in patients whose HbA1c records were available. This method was utilized as HbA1c has been proved to be effective in measuring the glycemic control in a total of the previous three months. Therefore, it gives a better judgment beyond the fluctuations that may occur as a result of depending only on the blood glucose levels. In this study, the prevalence rate of poor glycemic control was estimated to be 81.5%. This rate is higher than other reported rates within Saudi Arabia. Alzaheb et al. [16] reported a prevalence rate of 74.9%, Al-Rasheedi et al. [17] reported a rate of 67.7%, which is similar to the 67.9% reported by Badedi et al. [18], while Khan et al. [19] reported a rate of 74%. High prevalence rates were also reported in diabetes patients from other Middle Eastern countries, such as Oman (65.0%), Kuwait (78.8%), United Arab Emirates (69.0%), and Jordan (65.1%) [20, 21]. This sheds light on the high prevalence of poor glycemic control in the region, and therefore, investigations should be undertaken to find the associated risk factors.

The logistic regression analysis showed that older patients were more likely to have better glycemic control. Ahmed et al. [11] reported that glycemic control was better in patients older than 65 years. Additionally, the authors reported that a 3% increase in the rate of achieving glycemic control was associated with a one-year increase in a diabetic patient. The authors justified their results by saying that older patients are usually assisted by other younger relatives [22]. Additionally, previous studies did not find any significance in the association between age and poor glycemic control [16]. Almetwazi et al. [23] even reported that older age was associated with higher rates of poor glycemic control due to the presence of co-morbidities and suggested that follow-ups should be conducted for these patients regularly.

Another associated factor was found to be the duration of the disease. Ahmad et al. [11] found that a rate of 5% reduction in glycemic control was associated with a one-year increase in the duration of having DM. These results are consistent with the results of previous studies in the literature, which showed that having DM for longer durations negatively impacts glycemic control [24]. This can be explained by the fact that β-cell dysfunction progresses with time, which increases the need to adjust the treatment regimens more regularly [25]. Another factor that was found to be associated with poor glycemic control is the combined use of insulin and oral drugs. This is consistent with the result found by Ahmad et al. [11]. Previous reports have also reported that a triple therapy of anti-diabetic drugs was an effective regimen in glycemic control [11, 26]. On the other hand, previous investigations reported that insulin alone or combined with oral antidiabetic agents significantly produces better glycemic control results [27, 28]. However, when using regimens such as these, frequent follow-ups and monitoring should be conducted to ensure the appropriate optimal doses are prescribed and increase the patients’ compliance.

In addition, we did not find any significance in terms of patients’ sex to be associated with poor glycemic control, which is consistent with the results of previous research [11, 18]. On the other hand, elevated HbA1c levels were reportedly more associated with female than male patients in Abudawood et al. [29]. Although previous reports have also found obesity, high LDL levels, and associated comorbidities to be associated with poor glycemic control, in this study and other studies, no association between BMI, LDL levels, comorbidities, and the poor glycemic effect was found [11].

A cause/effect association could not be done in this study due to the limitations of the study design. Another limitation is the self-reporting of some variables, such as tobacco use, which may have been subject to subjective or denial bias. Furthermore, drug compliance was not determined due to its subjective understanding from one patient to another.

## Conclusions

The results of this study indicate the high prevalence rate of poor glycemic control among Saudi patients, which was higher than shown in previous reports. We also found that old age was associated with better glycemic control. On the other hand, longer durations of having diabetes, and the combined use of insulin and oral drugs, was associated with poor glycemic control. In addition, neither BMI, LDL levels, gender, or the occurrence of complications showed significant associations with poor glycemic control. More interest should be given to awareness programs promoting disease among older age groups as they are more prone to have poor glycemic control than younger patients.
